# The impact of image dynamic range on texture classification of brain white matter

**DOI:** 10.1186/1471-2342-8-18

**Published:** 2008-12-23

**Authors:** Doaa Mahmoud-Ghoneim, Mariam K Alkaabi, Jacques D de Certaines, Frank-M Goettsche

**Affiliations:** 1 United Arab Emirates University, Faculty of Science, Physics Department, AlAin, United Arab Emirates; 2 UPRES-EA 3890 Imagerie Fonctionnelle et Vectorisation en Cancérologie, IFR 140 GFAS, Rennes, France; 3 Institute of Meteorology and Climate Research, Forschungszentrum Karlsruhe, Karlsruhe, Germany

## Abstract

**Background:**

The Greylevel Cooccurrence Matrix method (COM) is one of the most promising methods used in Texture Analysis of Magnetic Resonance Images. This method provides statistical information about the spatial distribution of greylevels in the image which can be used for classification of different tissue regions. Optimizing the size and complexity of the COM has the potential to enhance the reliability of Texture Analysis results. In this paper we investigate the effect of matrix size and calculation approach on the ability of COM to discriminate between peritumoral white matter and other white matter regions.

**Method:**

MR images were obtained from patients with histologically confirmed brain glioblastoma using MRI at 3-T giving isotropic resolution of 1 mm^3^. Three Regions of Interest (ROI) were outlined in visually normal white matter on three image slices based on relative distance from the tumor: one peritumoral white matter region and two distant white matter regions on both hemispheres. Volumes of Interest (VOI) were composed from the three slices. Two different calculation approaches for COM were used: i) Classical approach (CCOM) on each individual ROI, and ii) Three Dimensional approach (3DCOM) calculated on VOIs. For, each calculation approach five dynamic ranges (number of greylevels N) were investigated (N = 16, 32, 64, 128, and 256).

**Results:**

Classification showed that peritumoral white matter always represents a homogenous class, separate from other white matter, regardless of the value of N or the calculation approach used. The best test measures (sensitivity and specificity) for average CCOM were obtained for N = 128. These measures were also optimal for 3DCOM with N = 128, which additionally showed a balanced tradeoff between the measures.

**Conclusion:**

We conclude that the dynamic range used for COM calculation significantly influences the classification results for identical samples. In order to obtain more reliable classification results with COM, the dynamic range must be optimized to avoid too small or sparse matrices. Larger dynamic ranges for COM calculations do not necessarily give better texture results; they might increase the computation costs and limit the method performance.

## Background

Automated statistical and structural methods applied to digital medical images have shown that the amount of quantitative information available in the image exceeds the capacity of the human visual system [1]. These methods assume that image greylevel relationships and spatial distribution are directly influenced by the properties of the underlying tissue, which themselves, are dynamic and depend on biological and chemical composition. Therefore, minor image modifications can be quantified and monitored by appropriate methods even before they are perceivable by the human eye. Such automated methods are collectively known as Texture Analysis (TA) [1].

Since the physical properties of tissues are the basis for operating imaging modalities, the reliability of the imaging output depends on the ability of the modality to provide contrast between different tissues as well as local contrast that shows early changes in the physical-chemical properties within that tissue. MRI is known to provide the best image contrast among the imaging modalities available so far; therefore, MR images are believed to be rich in digital information that can be exploited by TA and would be of important analytical and diagnostic utility. In recent years, Texture Analysis on Magnetic Resonance Images (MRI-TA) has been applied successfully in clinical and experimental studies and is regarded as a reliable noninvasive tool of investigation, which combines the high contrast of MRI with the good sensitivity and specificity of TA. The quantitative texture data obtained from TA are relative rather than absolute; therefore, MRI-TA usually has to be followed by a standard classification method.

It has been demonstrated with laboratory animals that *in-vivo *MRI-TA of muscles correlates with histology during degeneration and regeneration processes [2]. Direct relationships between muscle contents of fat and collagen were found using texture classification on high resolution MRI [3]. MRI-TA has been clinically investigated on several tissues such as breast lesions [4], and hepatic fibrosis [5]. Brain tissue also has been studied using MRI-TA [6-8]. These studies recommended MRI-TA as a potential tool for non-invasive investigations of cerebral tumors as well as for healthy white and grey matter. In a previous work on brain gliomas, we investigated peritumoral white matter in regions defined by the radiologists as normal nonpathological tissues, but which were in the proximity of visible tumor margins. MRI-TA classified these regions as a homogenous texture class, separate from the other white matter regions which clustered in one broad class [8]. We suggested that this different texture could be due to invisible proliferation by tumoral cells [8].

Since TA is based on calculations with image greylevels, it becomes crucial to understand the impact of changing image properties on the stability of texture results and on the performance of the method [9]. Investigations of such relationships eventually will lead to an optimized and more reliable method for biomedical image analysis applications, which will require less processing time and less extensive calculations.

It is commonly assumed in TA applications that increasing the image dynamic range on which texture is evaluated improves textural feature representation; and consequently, gives better classification results. However, there is no evidence in biomedical image analysis literature to confirm or reject this assumption. The objective of the current work is to investigate the dependence of a commonly used TA method, the Cooccurrence Matrix (COM), on image dynamic range and matrix calculation approach for classifying white matter regions.

## Methods

### Patients and MRI data

In agreement with the French ethical legislation on clinical trials, whole brain MRI datasets were acquired in the sagittal plane for ten Glioblastoma patients (age = 53 ± 18; histologically confirmed by biopsy) using a Philips 3-T Achieva MR system (Philips Medical System, Best, Netherlands). The imaging sequence used was Three-Dimensional Gradient Echo (TR = 9.87 ms, TE = 4.56 ms, flip angle = 8°). Field of View (FOV) = 256 mm × 256 mm, matrix size = 256 × 256, and a slice thickness of 1 mm, gave isotropic voxel resolution of 1 mm^3^. Transversal sections were reconstructed from the original sagittal plane. Imaging procedures and clinical diagnosis were performed in Rennes University Hospitals, Rennes, France.

**Figure 1:**
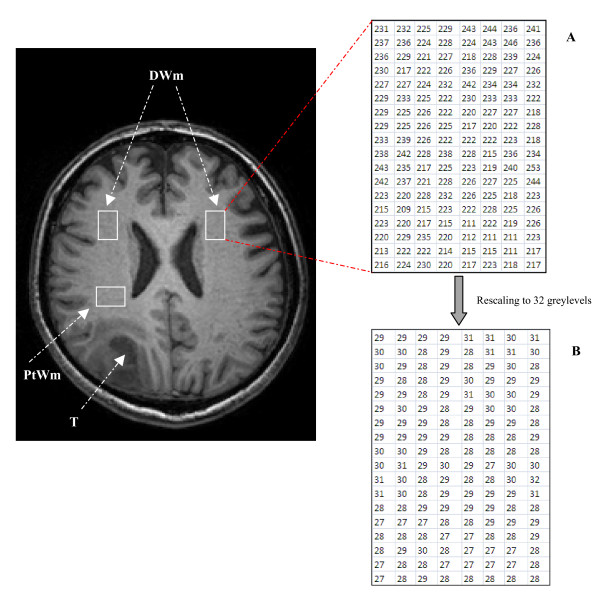
**MR image of brain glioblastoma and the surrounding white matter**. Transversal slice of MRI of brain glioblastoma showing Tumor, T; and the normal white matter regions (solid lines): PtWm, Peritumoral; and DWm, Distant White matter. An ROI and the corresponding matrix are linked (red dashed lines) to illustrate the rescaling process. Matrix **A **represents the original ROI which has a dynamic range from 0 to 255 greylevels. The matrix **B **shows the same ROI after multiplying each pixel with the ratio of the maximum greylevel value allowed in **B **(31 in this case) to the actual maximum greylevel value of **A**.

Each patient showed a tumor mass developed within the brain white matter. Three Regions of Interest (ROI)s were manually outlined in the normal white matter by a radiologist on a first transversal image Slice (S1) according to relative distance of the region to the tumor: one Peritumoral White matter (PtWm) close to the visible margins of the tumor; and two Distant White matter (DWm) taken far from the tumor on both hemispheres (figure 1). Each ROI contains of about 100–200 pixels. Volumes of Interest (VOI)s were constructed by copying the ROI position to the next two adjacent transversal slices (S2 and S3) producing volumes of about 300–600 voxels each. The VOI boundaries were inspected carefully to avoid overlapping structures. Only for one patient the location of the tumor did not allow for outlining a PtWM. A total number of 89 ROIs and 29 VOIs were available for this study.

### Cooccurrence Matrices

The Cooccurrence Matrix (COM) was first introduced by Haralick [10] along with 14 derived features; most of them quantitatively describe image homogeneity and greylevels correlations. Some COM features have been found to be discriminative, and therefore, can be used for texture classification [10]. In a digital image, the number of bits-per-pixel (bp) coding determines the maximum number of greylevels (N) in the image (2^bp ^= N). Hence, the allowed dynamic range of greylevels is from 0 to (N-1).

The Classical approach of COM calculation (CCOM) samples the probability density function *P*_*d*,*θ*_*(i, j)*, which gives the probability of finding the two greylevels *i *and *j *at a distance *d *(*d *= 1,2,3,...) in the direction of angle *θ*_*xy *_(*θ*_*xy *_= 0°, 45°, 90°, and 135°), on a two dimensional image defined on the *x*- and *y*- axes. This calculation approach ignores useful spatial information that can be obtained from relationships between slices. Therefore, recent approaches try to maximize the usefulness of COM by including data at various angles on the *z*-axis. One of these approaches is known as Three Dimensional Cooccurrence Matrix (3DCOM) [8]. 3DCOM is calculated on image volumes composed of several adjacent slices, and involves nine angles on the *z*-axis (*θ*_*z *_= 0°, 45°, 90°, 135°, 180°, 225°, 270°, 315°, and co-linear) in addition to angles *θ*_*xy*_. More details can be found in [8]. A Direction Independent (DI) matrix results from summing COM over all angles. This indicated in the notation below by *θ *= DI.

In this work, both approaches are calculated: i) CCOM on ROIs of the three adjacent slices (S1, S2, S3) giving three matrices CCOM-S1, CCOM-S2, and CCOM-S3, respectively; and ii) 3DCOM on the VOI given by the three slices. For both approaches, the resulting matrix is always symmetric about its diagonal and of NxN size with N^2 ^number of entries. Five parameters were calculated from each matrix: Angular Second Moment, Inverse Difference Moment, Entropy, Contrast and Correlation [10]. These five parameters were selected because they were found to be good descriptors of white matter texture in a previous work [8]. They provide the main information about image homogeneity and the existence of correlated patterns in the image.

The original MR images are usually digitized over 16 bits-per-pixels (65536 greylevels). It is computationally extensive and time consuming to calculate COM over such a large dynamic range. Therefore, it is a standard procedure in medical image analysis to apply a quantization process in order to reduce the original range to a user-defined value of N. This is done by scaling the original pixel values with the ratio between the maximum greylevel allowed in the rescaled image and the actual maximum greylevel in the original image (figure 1). Prior to COM calculations, each ROI is rescaled for five different values of N, (N = 16, 32, 64, 128, and 256). All texture calculations and image processing methods were implemented using Matlab^® ^(ver 7.0, Math-Works Inc., Natick, MA, USA), on a PC with Intel^® ^Pentium^® ^4.0 processor and 1.24 Gb RAM.

### Features Selection and Classification

Features selection aims to identify the most discriminating parameters from each matrix that separate the different classes most efficiently. Fisher-coefficient (*F*-coefficient) was calculated for this purpose, giving the ratio of *between class variance *to *within class variance *[11] for each parameter. The ten parameters of the highest *F*-coefficient were entered to Linear Discriminant Analysis (LDA) for classification. LDA aims to find a linear transform matrix such that the ratio of within-class scatter matrix to between-class scatter matrix is maximized. Such a transform is composed of eigenvectors corresponding to the largest eigenvalues of this ratio of matrices; more details about the classification method can be found in [12]. Cross validation was performed using "leave-one-out" criterion, which works by leaving one observation (i.e one ROI) out of the classifier each time the classification model is recalculated and then project this observation into the model to test its validity. This process is carried out for all observations. The percentages of False Negatives (FN) and False Positives (FP) were evaluated. The Receiver Operating Characteristic (ROC) curve was analyzed, which represents the relationship between the '1-Specificity' and the 'Sensitivity' of the test. The Area Under the ROC Curve (AUC) is used to judge the separability of the two classes for the given dataset and classifier. An AUC of 1.0 represents a perfect classifier, while an AUC of 0.5 represents a random classifier.

Features Selection was performed using B11 software (version 3.2, ^©^1999–2002 by Michal Strzelecki), which is developed under the auspices of COST action B11 European project [12]. Linear Discriminant Analysis (LDA) was followed by cross validation and was performed using the software Minitab 15 (^© ^2007 Minitab Inc). The ROC curve was analyzed and AUC were calculated using SPSS 15.0 (^© ^1989–2006 SPSS Inc).

## Results and discussion

LDA classification on PtWm and DWm white matter regions always separated PtWm into a distinct homogenous class. This class was well distinguished for small as well as for large dynamic ranges for all matrices. However, the number of classification errors between the two classes depended remarkably on the dynamic range along with COM approach used. Table 1 represents the percentage of False Negatives (FN: PtWm classified as DWm) and False Positives (FP: DWm classified as PtWm) for each number of greylevels N and matrix calculation approach. The average errors and standard deviation (Mean ± SD) for CCOM-S1, CCOM-S2, and CCOM-S3 over the three slices is also presented and will be used for comparison with 3DCOM.

**Table 1:** Classification results using cross-validated LDA and for Peritumoral White matter (PtWm) classified as Distant White matter (DWm) (False Negative: FN).

	**16-GL**			**32-GL**			**64-GL**			**128-GL**			**256-GL**		
	
	**FN%**	**FP%**	**AUC**	**FN%**	**FP%**	**AUC**	**FN%**	**FP%**	**AUC**	**FN%**	**FP%**	**AUC**	**FN%**	**FP%**	**AUC**
**CCOM-S1**	22.00	15.00	0.82	33.00	5.00	0.81	33.00	10.00	0.785	11.00	5.00	0.915	22.00	15.00	0.815
**CCOM-S2**	55.00	25.00	0.60	25.00	44.00	0.655	33.00	20.00	0.735	33.00	10.00	0.785	22.00	10.00	0.84
**CCOM-S3**	33.00	20.00	0.735	33.00	10.00	0.785	11.00	15.00	0.87	11.00	10.00	0.895	22.00	10.00	0.84
**Mean ± SD**	**36.67 **± *16.80*	**20.00 **± *5.00*	**0.715**	**30.33 **± *4.62*	**19.67 **± *21.22*	**0.75**	**25.67 **± *12.70*	**15.00 **± *5.00*	**0.8**	**18.33 **± *12.70*	**8.33 **± *2.89*	**0.87**	**22.00 **± *0.00*	**11.67 **± *2.89*	**0.83**
**3DCOM**	22.00	10.00	0.84	22.00	20.00	0.79	33.00	10.00	0.785	11.00	10.00	0.895	44.00	5.00	0.755

DWm classified as PtWM (False Positive: FP); using five dynamic ranges (N = 16, 32, 64, 128, and 256). FN and FP are represented as percentage errors. AUC for each ROC curve is also demonstrated.

CCOM: Classical Cooccurrence Matrix calculated on slices: -S1, -S2, and -S3.

3DCOM: Three Dimensional Cooccurrence Matrix.

Mean ± SD the average and standard deviation of results for CCOM-S1, CCOM-S2, and CCOM-S3.

GL: Greylevels.

LDA: Linear Discriminant Analysis.

AUC : Area Under the Receiver Operating Characteristic (ROC) Curve.

Analyzing table 1 shows that the Mean FN or FP of CCOM (-S1,-S2,-S3) decreases progressively with increasing N until reaching N = 256 for which it increases again (table 1). For 3DCOM, the lowest value of FN occurs at N = 128, which was less than those obtained from Mean CCOM for any other N. The most discriminating parameters for this analysis and their *F*-Coefficients are presented in table 2. The percentage of FN shows a considerable increase when 3DCOM is calculated for N = 256; however, FP represents the lowest percentage obtained (table 1).

**Table 2:** The ten most discriminating parameters, according to the Fisher (*F*-) coefficient, between the two white matter classes (Peritumoral white matter and distant white matter).

**Most Discriminating Parameters**	***F*-Coefficient**
Entropy_*θ *= 0°	3.0972
Angular Second Moment_*θ *= 135°	2.2651
Entropy_*θ *= DI	1.9090
Entropy_*θ *= 135°	1.8852
Angular Second Moment_*θ *= 0°	1.8002
Angular Second Moment_*θ *= 45°	1.7164
Angular Second Moment_*θ *= 90°	1.6279
Angular Second Moment_*θ *= DI	1.5740
Entropy_*θ *= 45°	1.4461
Contrast_*θ *= DI	1.0305

Using Three-Dimensional Cooccurrence Matrix (3DCOM) for a number of greylevels N = 128.

DI: Direction Independent

*θ*: The angle of the parameter.

**Figure 2:**
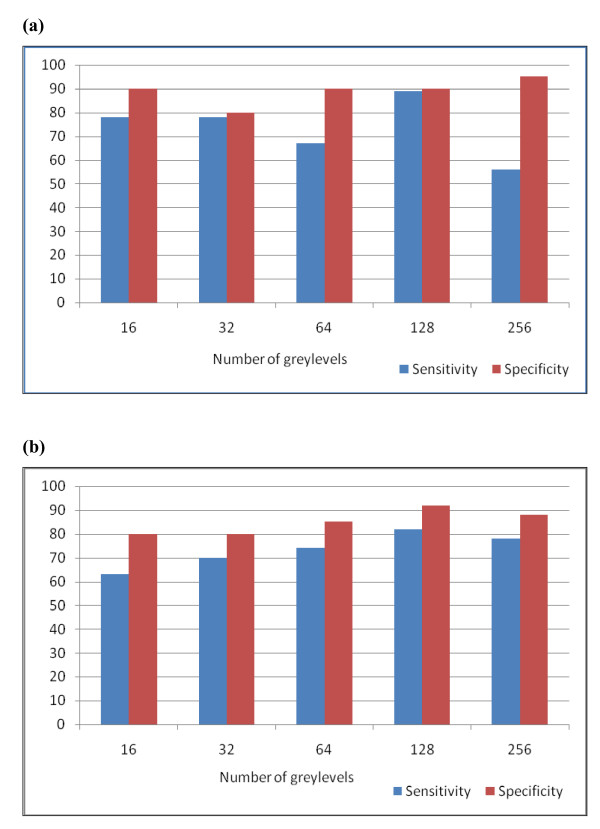
**Sensitivity and specificity bar graphs**. Sensitivity and specificity bar graphs for (a) 3DCOM on white matter VOIs; and, (b) The Mean value of (CCOM) on the individual slices ROIs (-S1, -S2, and -S3). CCOM: Two Dimensional Classical Cooccurrence Matrix. 3DCOM: Three Dimensional Cooccurrence Matrix. VOI: Volume of Interest. ROI: Region of Interest.

**Figure 3:**
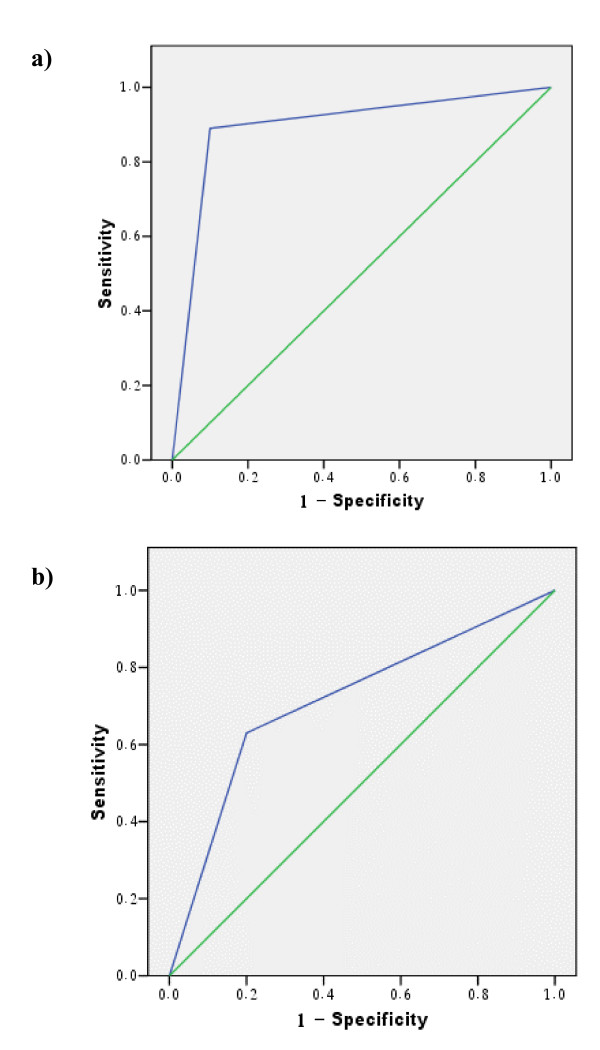
**ROC curves showing the highest and lowest AUC**. Receiver Operating Characteristic (ROC) curves showing: a) the highest Area Under the Curve (AUC) (= 0.895) which was obtained using 3DCOM at N = 128; and, b) the lowest AUC (= 0.715) obtained using the Mean CCOMs at N = 16. CCOM: Two Dimensional Classical Cooccurrence Matrix. 3DCOM: Three Dimensional Cooccurrence Matrix.

The bar graph of test outcomes measures (sensitivity and specificity) (figure 2) demonstrates a balanced tradeoff between the sensitivity and specificity of the 3DCOM method at N = 128 and N = 32 with higher values at the former (figure 2a). This balance is lost at other values of N (figure 2b). The Mean CCOM method on the three slices (-S1,-S2,-S3) shows the highest sensitivity and specificity at N = 128 (figure 2b). For either CCOM or 3DCOM, figure 2 demonstrates that the specificity of the method is always higher than its sensitivity. The Area Under the ROC Curve (AUC) represents a comprehensive measure for evaluating the accuracy of the classifier (table 1). By comparing AUCs of the Mean value of the three CCOMs and those of 3DCOM, it can be shown that the highest AUC value was obtained for 3DCOM at N = 128, while the lowest was obtained for Mean CCOMs at N = 16 (figures 3a and 3b, respectively). It can also be shown that the highest value of AUC among Mean CCOMs was obtained also at N = 128 (table 1).

In this study, PtWm clustering as a separate white matter region is consistent with previous findings [8]; however, we demonstrate in the current work that classification accuracy is highly dependent on the dynamic range of image quantization for both COM calculation approaches (CCOM and 3DCOM). Also, we can see that classification results among different slices might give diverse results in spite of carrying out the analyses on identical positions. This can be demonstrated for FN at N = 16 that gave 22% on CCOM-S1 and 55% on CCOM-S2.

It can be also shown that calculating 3DCOM on small dynamic ranges (N = 12, 32 and 64) does not enhance classification as long as the dynamic range remains relatively small. In contrast, 3DCOM on a larger dynamic range (N = 128) enhances classification remarkably, but a further increase of N worsens the method's sensitivity. Although method's specificity has increased at N = 256, the tradeoff between sensitivity and specificity remains an important factor to take into account when evaluating the method's performance. Therefore, N = 256 is probably not a good choice for 3DCOM analysis.

The relationship between the dynamic range and classification accuracy can be related to COM characteristics. This matrix, by definition, is a probability density matrix of unit sum. Decreasing the dynamic range means that the original ROI will be reduced to smaller adjoining values of greylevels as shown in figure 1; therefore, cooccurrence matrix will be smaller and the joint probabilities will be estimated for a limited number of matrix entries (eg. N = 16, COM size = 16 × 16 = 256 entry). This could be insufficient to represent texture features and may result in higher classification errors. On the other hand, increasing the dynamic range will spread the greylevels over a larger scale producing matrices with sufficient number of entries; and then, discriminating texture features would have more chance to appear; consequently, this would reduce the percentage error. Further increase of N values produces sparse matrices with probabilities broken down over a huge number of entries (65535 for N = 256); in other words, feature representation would be weakened and classification errors increased. It merits to mention that the processing time for calculating 3DCOM using N = 128 was within a fraction of a second, while it took almost 30 seconds for calculating the same matrix using N = 256. The increase in processing time is even more significant for larger VOIs.

From these results, we recommend quantizing image ROI/VOI to a number of N = 128 greylevels prior to texture analysis of white matter. This value represents a compromise for applying cooccurrence matrix calculations in white matter texture studies for the two dimensional approach as well as for the three dimensional one.

## Conclusion

In this work we have demonstrated that the dynamic range on which texture features are evaluated, particularly when using the cooccurrence matrix, can directly influence the accuracy of classification of white matter regions. We found that rescaling the ROI to a dynamic range of greylevels from 0 to 127 (i.e. N = 128) gives the best classification results using two dimensional cooccurrence matrix CCOM represented by the mean value of the three slices (S1, S2, and S3). It also gives the best balance between sensitivity and specificity, using the three dimensional cooccurrence matrix 3DCOM. For both types of matrices, the AUC of the ROC curve was maximum at N = 128. We conclude that a reduced user-defined dynamic range can be faster, computationally less extensive, and more efficient in separating texture classes.

## Competing interests

The authors declare that they have no competing interests.

## Authors' contributions

DMG has designed the study, implemented the texture analysis methods on Matlab^® ^7.0, acquired data, analyzed results, and drafted the manuscript. MKA has participated in the programming procedures. JDC has set and supervised the protocol of MR image acquisition in Rennes University Hospitals according to rules and regulations set by Ethics Committees. FMG has participated in results analysis and critical revision of the manuscript.

## Pre-publication history

The pre-publication history for this paper can be accessed here:


